# Faceted polymersomes: a sphere-to-polyhedron shape transformation†
†Electronic supplementary information (ESI) available. See DOI: 10.1039/c8sc04206c


**DOI:** 10.1039/c8sc04206c

**Published:** 2019-01-10

**Authors:** Chin Ken Wong, Adam D. Martin, Matthias Floetenmeyer, Robert G. Parton, Martina H. Stenzel, Pall Thordarson

**Affiliations:** a School of Chemistry , University of New South Wales , NSW 2052 , Australia . Email: m.stenzel@unsw.edu.au ; Email: p.thordarson@unsw.edu.au; b ARC Centre of Excellence in Convergent Bio-Nano Science and Technology , Australia; c Centre for Advanced Macromolecular Design (CAMD) , School of Chemistry , University of New South Wales , Sydney , NSW 2052 , Australia; d Centre for Microscopy and Microanalysis , The University of Queensland , St. Lucia , Brisbane , QLD 4072 , Australia; e Institute of Molecular Bioscience , The University of Queensland , St. Lucia , Brisbane , QLD 4072 , Australia

## Abstract

The creation of “soft” deformable hollow polymeric nanoparticles with complex non-spherical shapes *via* block copolymer self-assembly remains a challenge. In this work, we show that a perylene-bearing block copolymer can self-assemble into polymeric membrane sacs (polymersomes) that not only possess uncommonly faceted polyhedral shapes but are also intrinsically fluorescent. Here, we further reveal for the first time an experimental visualization of the entire polymersome faceting process. We uncover how our polymersomes facet through a sphere-to-polyhedron shape transformation pathway that is driven by perylene aggregation confined within a topologically spherical polymersome shell. Finally, we illustrate the importance in understanding this shape transformation process by demonstrating our ability to controllably isolate different intermediate polymersome morphologies. The findings presented herein should provide opportunities for those who utilize non-spherical polymersomes for drug delivery, nanoreactor or templating applications, and those who are interested in the fundamental aspects of polymersome self-assembly.

## Abstract 2

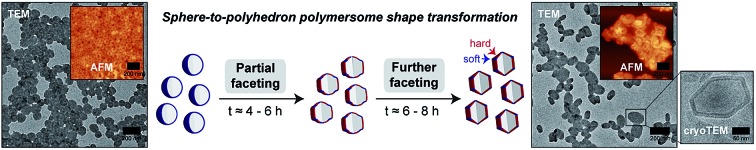
We uncover how our polymersomes facet through a sphere-to-polyhedron shape transformation pathway that is driven by perylene aggregation confined within a topologically spherical polymersome shell.

## 

Over the past few decades, the field of inorganic nanoparticle synthesis has made tremendous progress, to the point where it has now become routine to bottom-up assemble “hard” metal nanoparticles with a plethora of non-spherical shapes.^1^–^9^ Intriguingly though, the self-assembly of “soft” dynamic and deformable non-spherical polymer nanoparticles still remains a challenge even today.^10^

Among the many types of self-assembled polymer nanoparticles described in the literature, hollow polymeric membrane sacs known as polymer vesicles (polymersomes) exhibit great structural versatility, and have in recent years been shown to be capable of being subjected to some shape transformation processes. Typical examples of non-spherical polymersome shapes^11^ that have been reported so far include stomatocytes,^12^–^14^ strongly segregated two-faced Janus,^15^,^16^ tubes^17^–^19^ and ellipsoids.^19^–^23^ More complex vesicular shapes such as polyhedrons have also been shown to be accessible *via* computational modelling,^24^–^27^ but rarely so experimentally with polymers.^28^,^29^ Considering that recent progress in polymersome shape control has been largely driven by the desire to control their macroscale function for many emergent applications (*e.g.*, in the fields of nanomotors/nanoreactors,^30^–^33^ drug delivery^18^,^34^–^36^ and confined crystallization^37^), there is a clear need to advance understanding in this area of study. Taking the drug delivery field as an example, where cellular uptake efficiency has been shown to vary significantly between nanoparticles of different shapes and forms,^38^,^39^ there are clearly many exciting opportunities for the use of non-spherical polymersomes.

Unlike morphological changes that are commonly seen in block copolymer systems (*e.g.*, micelles-to-rods or rods-to-lamellae), the shape transformation of spherical polymersomes require an added level of complexity on top of the typical parameters that govern block copolymer self-assembly such as block length and composition, block–block interactions and block–solvent interactions.^40^ Generally speaking, in order generate non-spherical polymersomes, one would first have to select an appropriate combination of block copolymer and solvent system that specifically targets the spherical polymersome morphology. This would then have to followed by the introduction of an external factor or force (*e.g.*, osmotic pressure, supramolecular interactions or liquid-crystallinity)^12^,^19^,^29^ that helps to initiate and direct the shape transformation process.

Recently,^19^ we reported on the controlled formation of non-spherical (ellipsoidal and tubular) polymersomes (Fig. 1A) *via* a solvent-controlled supramolecular approach that exploits the use of a carefully designed block copolymer bearing perylene diester monoimide side chains, PEG_43_-*b*-P(NIPAM_21_-*co*-PDMI_9_). Motivated by our earlier success in generating shape anisotropic polymersomes, we were curious to explore the possibility of extending our methodology to access more sophisticated non-spherical shapes (*e.g.*, polyhedrons). We envisaged that the use of a structurally similar block copolymer with an inherently higher rigidity may allow us to achieve the intended outcome above. Accordingly, we synthesized a block copolymer with almost doubled perylene diester monoimide side chain content, PEG_43_-*b*-P(NIPAM_23_-*co*-PDMI_19_) and indeed, as will be discussed below, this newly synthesized block copolymer was able to self-assemble into polyhedral polymersomes (Fig. 1B).

**Fig. 1:**
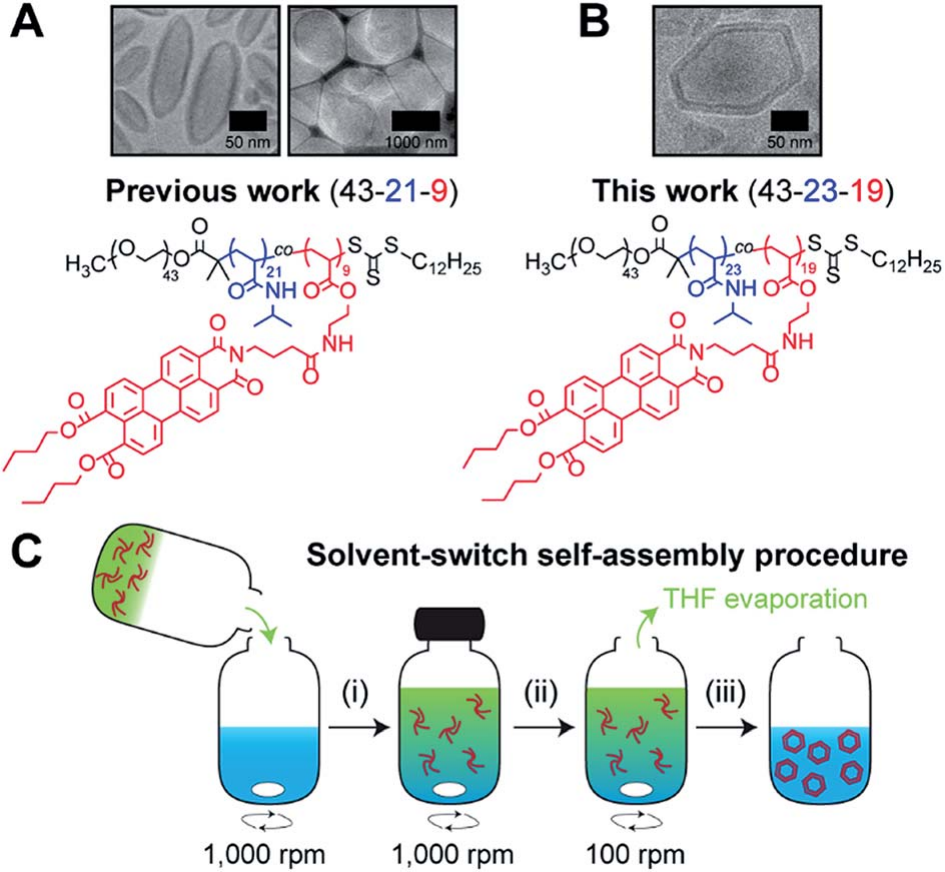
Chemical structure of perylene-bearing polymer used in (A) our previous work^19^ PEG_43_-*b*-P(NIPAM_21_-*co*-PDMI_9_) and in (B) this work PEG_43_-*b*-P(NIPAM_23_-*co*-PDMI_19_). Shown above each chemical structure are cryo-TEM images of our non-spherical polymersomes that are accessible *via* self-assembly. (C) Schematic depicting the solvent-switch process that was used to induce polymersome self-assembly. (i) Initial dissolution of polymer (red) in THF (green), and subsequent addition into water (blue); (ii) homogenization of polymer/THF/water mixture; (iii) slow removal of THF *via* evaporation to yield an aqueous polymersome solution. Note that the polymer already exists in an aggregated state in THF due to solvophobic effects and the strong aromatic stacking interactions between perylene moieties.

Herein, we report a detailed physical and chemical characterization of our faceted polymersomes, along with our findings on how faceting occurs through a sphere-to-polyhedron shape transformation pathway driven by perylene aggregation confined within a topologically spherical polymer shell. Lessons learned from this detailed study on the underpinning self-assembly mechanism will help to establish guidelines on what parameters govern the formation of polyhedrons in polymersome systems.

In order to induce the self-assembly of our polymersomes, we utilize a well-established protocol known as the solvent-switch method.^41^ Briefly, this process (Fig. 1C) involves (i) the dissolution of the polymer in THF (good solvent) and subsequent addition of the polymer/THF solution into water (non-solvent), (ii) a short homogenization step under vigorous stirring, and (iii) the removal of THF *via* slow evaporation under stirring to finally yield an aqueous polymersome solution. We should mention, based on our experience from earlier work,^19^ that while THF is a good enough solvent to solubilize the polymer in step (i), it is important to realize that the polymer does not exist as free unimer chains in solution, but rather as aggregates due to solvophobic effects and the strong aromatic (π–π) stacking interactions between perylene moieties on the polymer (Fig. S1†). Additionally, an evaporation time of 24 h in step (iii) effectively removes ∼99.9% of THF used for self-assembly (see Fig. S2† for quantification of residual THF).

As a starting point in this study, we began by investigating how solvent quality influenced the self-assembly of the faceted polymersomes' building block PEG_43_-*b*-P(NIPAM_23_-*co*-PDMI_19_). To this end, we varied the solvent quality used for self-assembly from 55–75% (v/v) THF/water (while maintaining the amount of water used at 500 μL, so in essence, only the volume of THF was changed) and visualized the resulting self-assembled structures using transmission electron microscopy (TEM) after complete evaporation of THF.

Interestingly, we found that faceted polymersomes could only be obtained when the THF/water mixture used was between 65% and 70% (Fig. 2A and C). When too little THF was used (*e.g.*, at 55% and 60% THF/water), rod-like micelles but no polymersomes were formed (Fig. S3A and B†). Likewise, at the highest THF/water ratio tested of 75%, no polymersomes formed. Instead, we observed the presence of large flower-like superstructures (Fig. S3C†). Evidently, the self-assembly of PEG_43_-*b*-P(NIPAM_23_-*co*-PDMI_19_) is strongly influenced by solvent quality. Dynamic light scattering (DLS) analysis performed on all five samples in aqueous solution doubly confirms the observed correlation between particle size and solvent quality (Fig. S3D and Table S1†).

**Fig. 2:**
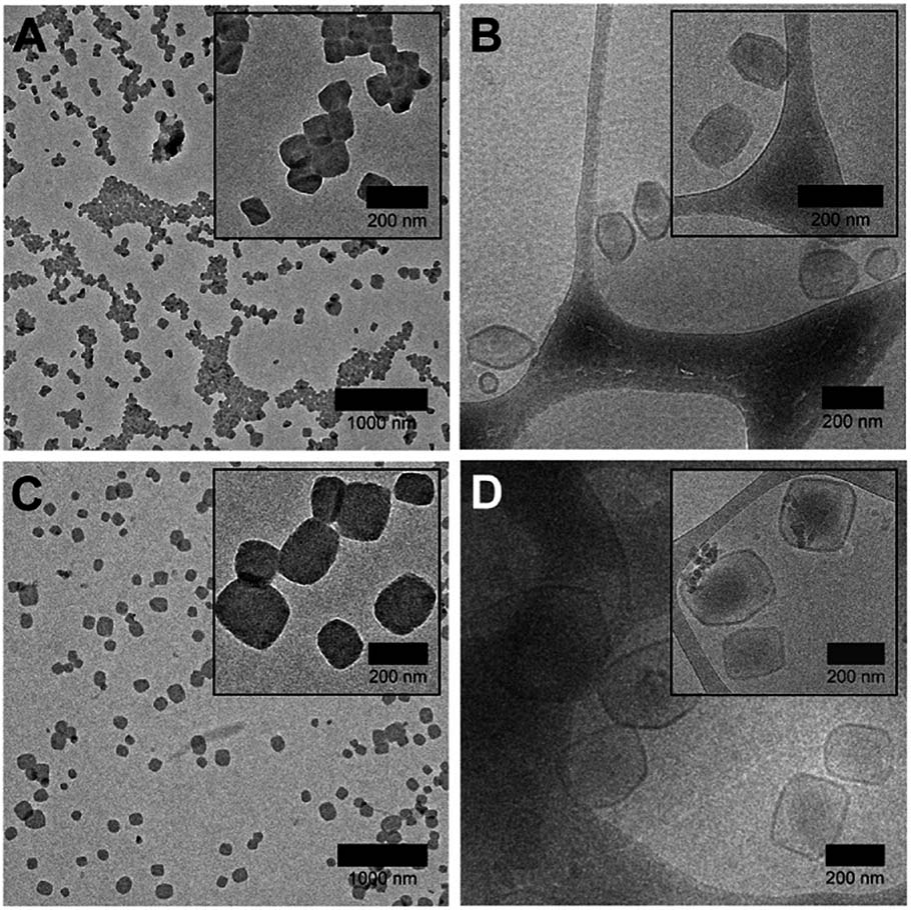
TEM (left) and cryo-TEM (right) images of faceted polymersomes prepared using the solvent-switch method at (A and B) 65% and (C and D) 70% THF/water. Higher magnification images are shown inset for clarity.

Next, we employed cryogenic-transmission electron microscopy (cryo-TEM) to better preserve and visualize the polymersome facets, which would typically otherwise be impaired by the drying process involved in conventional TEM sample preparation. The resulting cryo-TEM images of faceted polymersomes prepared at 65% and 70% THF/water are shown in Fig. 2B and D. In the vitrified state, the edges and vertices of the faceted polymersomes can be seen to be more well-defined, strongly contrasting their rounded appearance when viewed under conventional TEM. Conveniently enough, the polymersomes' membrane structure also became more apparent and easily discernible as dark outlines surrounding individual particles in the cryo-TEM images. Analysis of the cryo-TEM images using ImageJ^42^ revealed an average membrane thickness of ∼10 nm. Considering that the fully stretched chain length of PEG_43_-*b*-P(NIPAM_23_-*co*-PDMI_19_) is ∼10.6 nm, the measured membrane thickness suggests the existence of an interdigitated bilayer membrane structure in the faceted polymersomes.^43^

As can be seen from Fig. 2B, the majority of faceted polymersomes prepared at 65% THF/water exhibit a polyhedral shape resembling a hexagon with six edges and vertices. The faceted polymersomes prepared using 70% THF/water (Fig. 2D), on the other hand, appear more polydisperse and have 2D projected shapes such as tetragons (four edges and four vertices) and pentagons (five edges and five vertices). Some less defined, irregular polyhedrons can also be seen in this sample. Considering that the only parameter which was varied between the two samples analyzed (*i.e.*, 65% *vs.* 70% THF/water) is the THF amount used during self-assembly, it is thus apparent that the observed shape difference is related to the plasticizing nature of THF.

Having said all the above, it is important to realize that cryo-TEM only generates a 2D image projection of any 3D structure analyzed. Therefore, in this regard, the hexagonal-looking polymersomes (Fig. 2B), for instance, may in fact be a higher-dimensional polyhedron. To elaborate on this, a regular icosahedron with 20 faces, for example, can also appear to have only six edges or vertices when viewed top-down.^44^–^46^

To compensate for the projection limitation of cryo-TEM, we turned to cryogenic-electron tomography (cryo-ET), an advanced electron microscopy technique which allows structural analysis of macromolecules or macromolecular assemblies in their close-to-native state.^47^ In cryo-ET, a series of 2D images is first captured across a range of tilt angles, and then aligned using fiducial markers, and finally merged using computational algorithms to reconstruct a 3D representation of the analyzed sample. For our cryo-ET experiment, we chose to work with the faceted polymersomes prepared at 65% THF/water due to their shape uniformity and low polydispersity index (as confirmed by DLS; see Table S1†).

A resulting tomogram that displays a top-to-bottom sectional view of the sample is provided in the ESI (Movie S1†). To aid the 3D visualization of the faceted polymersomes, a 360° projection of the same tomographic reconstruction with added transparency are also provided (Movie S2†). For further clarity, six selected top-to-bottom x,y-slices from the tomogram is shown in Fig. 3. The cryo-ET data elucidates an important information about the faceted polymersomes; that is, they are irregular polyhedrons.

**Fig. 3:**
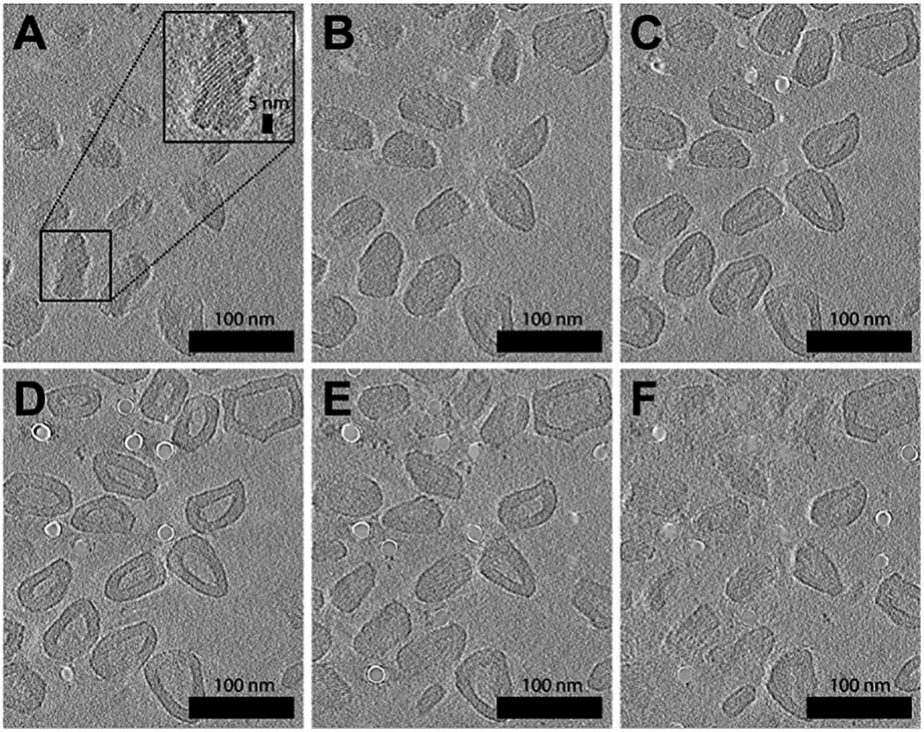
Cryo-ET images of (65% THF/water) faceted polymersomes. (A–F) Six top-to-bottom x,y-slices through the tomographic reconstruction. Shown inset in A is a higher magnification image that highlights the existence of smectic layers on the polymersome surface. A movie of the entire tomogram is provided as Movie S1 in the ESI.†

Another striking observation from the tomograms in Movie S1 and S2† is the existence of alternating dark and light domains (smectic layers) on the surface of the polymersomes (see also high magnification image in Fig. 3A inset), which suggests the confinement of a highly ordered lattice-like structure within the polymersome shell. The dark contrast stripes present can easily be attributed to the stacks of electron-deficient aromatic perylene units,^48^ while the light contrast stripes likely originate from the remaining segments of the polymer. To measure the smectic layer spacing, we performed fast Fourier transform (FFT) analysis (Fig. S4†) on different regions of the cryo-ET image shown in Fig. 3F. All four FFT patterns that were obtained revealed well-defined spots that correspond to a smectic periodicity of 2.2 nm.

Intrigued by our polymersomes' exotic appearance, we were next interested in unraveling the mechanisms that underlie the membrane faceting process. To achieve this, we again employed the use of TEM and cryo-TEM, as well as atomic force microscopy (AFM), to study the preceding formation of intermediate structures, and their eventual evolution into faceted polymersomes. For this experiment, small aliquots of polymer/THF/water mixture were sampled at different timepoints (*t* = 0.5, 1, 4, 6 and 8 h) during the THF evaporation step in the self-assembly process (refer back to (iii) in Fig. 1C for clarity) and deposited onto carbon-coated TEM grids. We would like to point out, though, that in this experiment, a slightly different THF/water volume ratio of 67.5% was used to generate moderately large but monodisperse faceted polymersomes for ease of imaging.

As can be seen from Fig. 4A, the early stage of self-assembly at around 0.5 h of THF evaporation seem to be quite complex, with the presence of large dense aggregates with an internal structure (see inset in Fig. 4A for a higher magnification image), as well as some surrounding lamellar-like structures. In order to better understand the observed morphologies, we again removed an aliquot from a freshly prepared polymer/THF/water mixture at the same evaporation timepoint (0.5 h) and analyzed it by cryo-TEM.

**Fig. 4:**
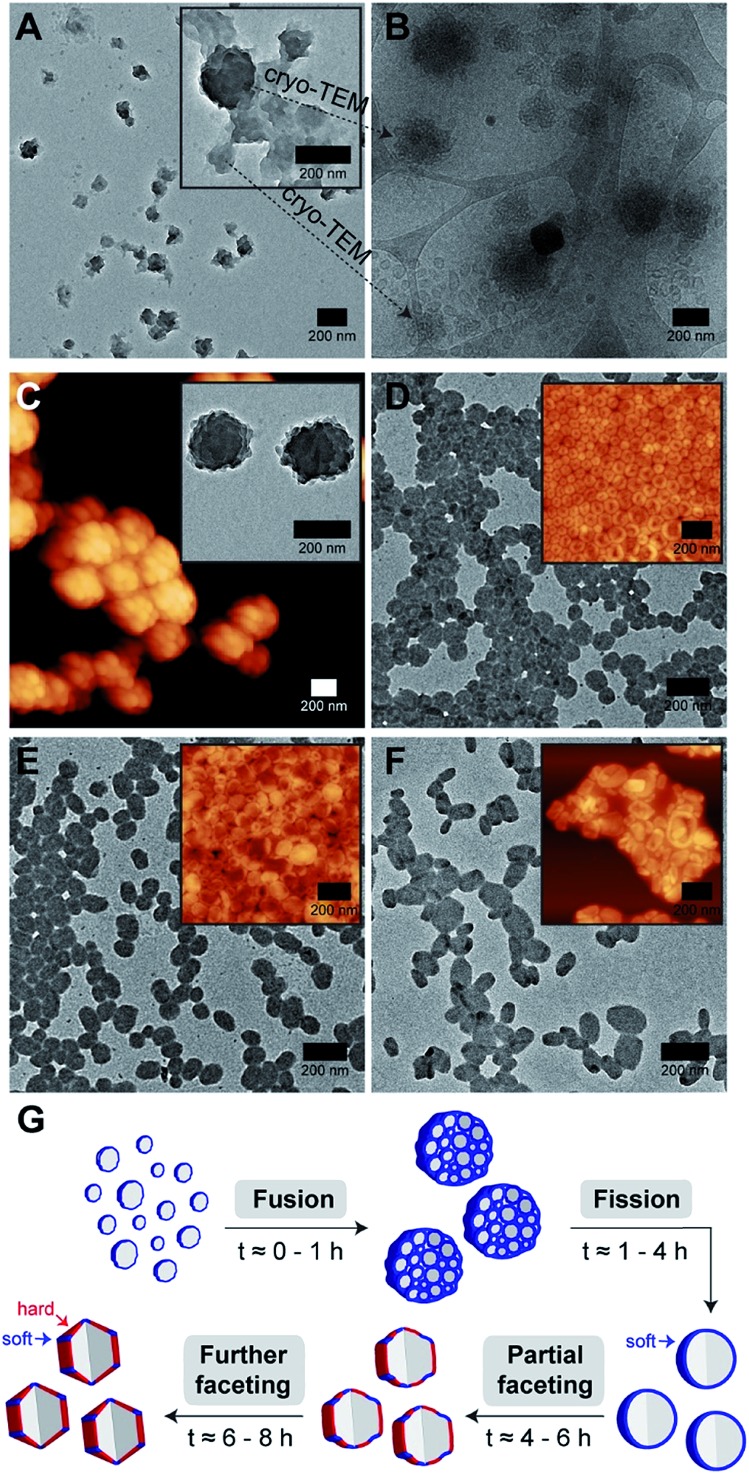
Kinetics of (67.5% THF/water) faceted polymersome formation evaluated using a combination of TEM, cryo-TEM and AFM following THF evaporation for (A and B) 0.5 h, (C) 1 h, (D) 4 h, (E) 6 h and (F) 8 h. (G) Proposed faceted polymersome self-assembly mechanism. The colors blue and red in G are used to highlight the soft and hard components present in each vesicular structure. A, D, E, F = TEM image; B = cryo-TEM image; C = AFM image; inset in C = TEM image; inset in D, E, F = AFM image.

Impressively, cryo-TEM (Fig. 4B) was able to better resolve the large dense aggregates and reveal that they are in fact large compound vesicles (LCVs).^49^–^51^ More fascinatingly, many irregularly shaped vesicles can also be seen in this sample (refer to bottom left corner of Fig. 4B), indicating that the formation of these LCVs is accompanied by some form of vesicle fusion or fission process. To probe this further, we carried out AFM and TEM characterization on an aliquot taken at a slightly later evaporation timepoint of 1 h (Fig. 4C). At this stage, we observed only the prevalence of LCVs, which hints that the formation of the LCVs is preceded by a fusion rather than fission process.

At the 4 h THF evaporation timepoint (Fig. 4D), LCVs could no longer be observed. Instead, we found the sample to be composed of purely spherical polymersomes. For this to happen, the LCVs must have undergone a subsequent fission process at some point in time between 1 and 4 h of the THF evaporation process. From 4 h onwards, the membrane faceting pathway was ultimately revealed. As can be seen from the TEM and AFM images in Fig. 4D–F, the polymersomes underwent a sphere-to-polyhedron shape transformation during this later stage of self-assembly. The polymersomes which were initially spherical at 4 h began to exhibit signs of partial faceting at 6 h, and subsequently transitioned into rigid polymersomes with defined facets at 8 h.

According to theory,^24^,^25^ closed faceted membrane structures such as the ones observed in this work, can be thought of simply as two-component shells that are composed of a hard and a soft component. The hard component, as represented by the color red in Fig. 4G, is responsible for the formation of rigid facets. The soft component, on the other hand, highlighted using the color blue in Fig. 4G, permits the formation of sharp vertices and edges by “gluing” individual facets together. Depending on the fraction of soft component relative to the hard component, the shell structure may take on a variety of shapes ranging from a soft sphere to a rigid polyhedron. Although the formation of extreme membrane curvature regions (*i.e.*, sharp vertices and edges) is typically very energetically costly, the shell structure is still able to adopt a polyhedral shape if there is enough favorable segregation of the lower-bending energy soft component around the curved regions.

Next, we asked the question – if we do consider our faceted polymersomes as two-component shell structures, what would be the factor that alters the balance between the soft and hard components? We know from our self-assembly kinetics experiment (Fig. 4) that our polymersomes undergo a sphere-to-polyhedron shape transformation during the later stages of self-assembly. Intuitively, this suggests that there is a progressive decrease in the polymersomes' soft component fraction over time (as evidenced by the loss of their isotropic spherical shape) that has to be related to the loss of plasticizing solvent THF from the polymersome system due to evaporation.

To answer our question above, we designed several experiments to study the role of THF in membrane faceting. In the first experiment, the intrinsic fluorescence of the polymersomes, which originates from the pendant perylene groups on the polymer building block, was exploited to study how THF causes nanoenvironmental changes within the polymersome membrane structure. To this end, a series of freshly prepared aqueous faceted polymersome solutions were doped with different amounts of THF (up to 20 vol% of THF in water), and subsequently characterized by fluorescence spectroscopy (Fig. 5A).

**Fig. 5:**
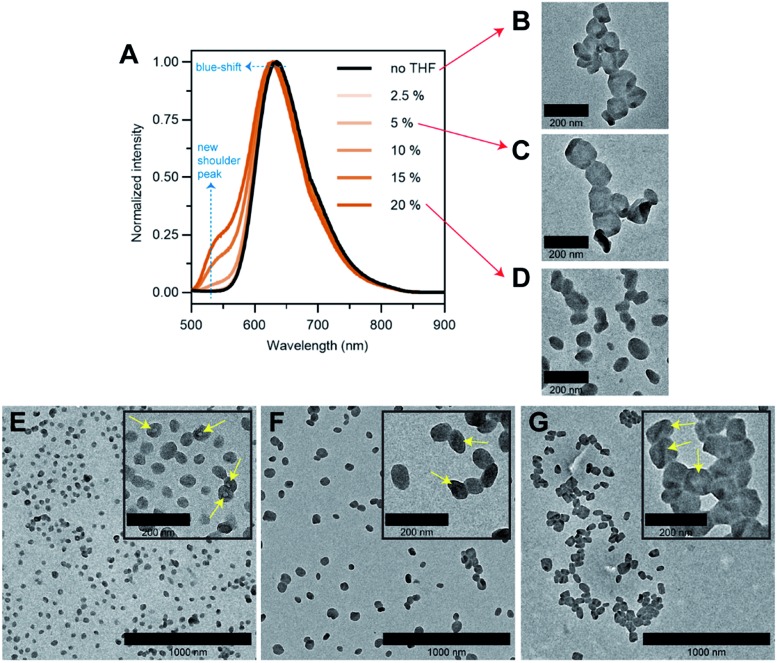
(A) Normalized fluorescence spectra of aqueous faceted polymersome solutions doped with different amounts of plasticizing solvent THF (0–20 vol%). *λ*_excitation_ = 480 nm. (B–D) TEM images of (65% THF/water) faceted polymersomes doped with (B) 0, (C) 5 and (D) 20 vol% of THF. (E–G) TEM images of quenched intermediate (E) spherical and (F) partially-faceted polymersomes, and the final product (G) faceted polymersomes. The yellow arrows in insets E–G highlight the hollow cores/membrane creases of the polymersomes. Successful quenching of the spherical and partially-faceted morphologies was achieved by dialyzing a 65% THF/water polymersome solution against water after a THF evaporation duration of 3.5 and 5 h, respectively. The faceted polymersomes in G were obtained after THF evaporation overnight.

In the absence of THF (black line, Fig. 5A), the faceted polymersomes exhibit a broad featureless fluorescence that peaks at 634 nm, which is characteristic of an excimer state formation within the H-aggregated perylenes that are stacked in a face-to-face fashion along a single polymer chain.^19^,^52^ When the faceted polymersomes were doped up to 5 vol% of THF, we observed little to no changes in the fluorescence profile. Interestingly, however, when the amount of doped THF was increased above 5 vol%, a shoulder peak centered around 545 nm began to appear (the origin of this peak is a direct reflection of an increase in the “softness” of the faceted polymersome membrane, as will be discussed further below). A slight blue-shift (∼5 nm) in fluorescence maxima was also observed in these samples. These observations are strong evidence that a reduction in intermolecular aromatic stacking interactions (*i.e.,* less aggregation) between individual perylene-bearing polymers within the polymersome membrane (due to solvation effects) is only observed when the amount of doped THF exceeds 5 vol%.

Next, we were interested to understand how changes in the extent of aromatic interactions through THF doping affected the polymersomes structurally. To study this, we performed TEM measurements on three faceted polymersome solutions that have respectively been doped with 0, 5 and 20 vol% of THF (Fig. 5B–D).

As one would expect, the negative control sample containing no added THF was found to consist of only polymersomes that look distinctly faceted (Fig. 5B). The second negative control sample which was doped with 5 vol% of THF, which according to the fluorescence study in Fig. 5A, exhibit no noticeable difference in aromatic interactions, should hypothetically consist of only faceted polymersomes. Indeed, this was found to be true, as can be seen from Fig. 5C. Finally, the sample doped with 20 vol% THF should, in theory, contain polymersomes that have undergone some form of nanostructural changes. Again, this was found to be true, as most polymersomes in this sample no longer exhibit well-defined faceted shapes (Fig. 5D). Instead, these polymersomes appear to adopt non-spherical shapes that bear significant structural resemblance to the partially-faceted polymersomes seen earlier in our self-assembly kinetics study (Fig. 4E). Taken together, both THF doping studies involving fluorescence spectroscopy and TEM unveil the crucial role of THF in facilitating facet formation in our system.

Finally, to accentuate the importance of realizing such a shape transformation process, we demonstrate the ability to selectively isolate two intermediate morphologies (spherical and partially-faceted polymersomes; Fig. 5E and F) in the self-assembly process, in addition to the final product (faceted polymersomes; Fig. 5G). To achieve this, we took advantage of our knowledge of the existence of the two intermediate morphologies at different THF evaporation timepoints during self-assembly (refer back to Fig. 4). In order to quench the intermediate spherical and partially-faceted polymersomes, we subjected two separate 65% THF/water polymersome solutions to extensive dialysis against water after THF evaporation durations of 3.5 and 5 h. As the removal of THF *via* dialysis occurs at a relatively higher rate than *via* evaporation under stirring, the intermediate structures were unable to undergo any further morphological transformation and instead become ‘frozen’ in kinetically-trapped states. As these intermediate structures are also intrinsically fluorescent, we intend to use them in a future comparative study to assess how polymersome shape influences their drug delivery capability.^39^

## Conclusions

In summary, we have presented here a detailed analysis on the formation of polyhedral faceted polymersomes with intrinsic fluorescence. Here, we have also for the first time experimentally visualized the entire sphere-to-polyhedron shape transformation process in polymersomes. Through a series of experiments, we further unveiled that the shape transformation proceeds due to the confined aggregation of perylenes within a topologically spherical polymersome shell. Lastly, we have shown here that the deciphering of such a shape transformation process enables us to selectively isolate intermediate polymersome morphologies. We foresee our faceted polymersomes being used as templates for the preparation of hollow faceted inorganic nanoparticles^53^ or for the construction of structural mimics of virus capsids,^54^ or as building blocks for creation of superlattices *via* shape-directed self-assembly.^55^,^56^

## Conflicts of interest

There are no conflicts to declare.

## Supplementary Material

Supplementary information

Supplementary movie

Supplementary movie
