# Supplementary material for: Faceted polymersomes: a sphere-to-polyhedron shape transformation
Source: Chem Sci. 2019 Jan 10;10(9):2725–31. doi: 10.1039/c8sc04206c (PMC6419931; doi:10.1039/c8sc04206c)
Supplement: Supplementary file 1 [file SC-010-C8SC04206C-s001.pdf]

Electronic Supplementary Material (ESI) for Chemical Science.
This journal is © The Royal Society of Chemistry 2019

Supporting Information

Faceted Polymersomes: A Sphere-to-Polyhedron Shape Transformation

Chin Ken Wong,[a,b,c] Adam D. Martin,[a,b] Matthias Floetenmeyer,[d] Robert G. Parton,[b,d]

Martina H. Stenzel,[a,c] and Pall Thordarson[a,b]

[a]School of Chemistry, University of New South Wales, NSW 2052, Australia.
[b]ARC Centre of Excellence in Convergent Bio-Nano Science and Technology, Australia.
[c]Centre for Advanced Macromolecular Design (CAMD), School of Chemistry, University of New South Wales, Sydney, NSW 2052, Australia.
[d]Institute of Molecular Bioscience, University of Queensland, St. Lucia, QLD 4072, Australia.

* To whom correspondence should be addressed.

Emails: m.stenzel@unsw.edu.au and p.thordarson@unsw.edu.au

Contents

Experimental Procedures

Materials....S2
Polymersome Self-assembly Procedure....S2
Transmission Electron Microscopy (TEM)....S2
Cryogenic-Transmission Electron Microscopy (cryo-TEM)....S2
Cryogenic-Electron Tomography (cryo-ET)....S2
Atomic Force Microscopy (AFM)....S3
Dynamic Light Scattering (DLS)....S3
Fluorescence Spectroscopy....S3
UV-Vis Spectroscopy....S3

Supplementary Figures and Tables
Figure S1 – Additional spectroscopy data....S4
Figure S2 – Gas chromatography-flame ionization detector (GC-FID) analysis....S5
Figure S3 –Additional TEM images and DLS results....S6
Figure S4 – Four fast Fourier transform (FFT) patterns from cryo-ET images....S6
Table S1 – Quantitative analysis of GC-FID data....S7
Table S2 – Solvent mixtures used for self-assembly and DLS results....S7
References....S7

Experimental Procedures

Materials

All chemicals were purchased from Sigma Aldrich and used as is, unless otherwise mentioned. Water used in experiments was dispensed from a Millipore Milli-Q Ultrapure Water System. TEM grids used were purchased either from ProSciTech (Australia) or Electron Microscopy Science (USA). The perylene-bearing polymer PEG_{43}-*b*-P($NIPAM_{23}$-*co*-$PDMI_{19}$) ($M_{n,NMR}$ = 18,259 g/mol; $M_{n,\,GPC}$ = 16,100 g/mol; $Đ$ = 1.09) was synthesized and characterized as per our previous report.[1]

Polymersome Self-Assembly Procedure

In a typical experiment, a 4 mL vial equipped with a magnetic stir bar (10 mm length × 5 mm width) was charged with 500 μL of water. To this solution was then added the required amount of THF (Table S2) containing 0.5 mg of PEG_{43}-*b*-P($NIPAM_{23}$-*co*-$PDMI_{19}$) in one portion. Following THF-to-water addition, the vial was immediately capped with a lid and the solution stirred at 1,000 rpm. After 30 min of vigorous stirring, the stir rate was reduced to 100 rpm, after which the lid was removed to allow the mixture to evaporate under ambient conditions for at least 24 h in a fume hood (note that the evaporation time may have to be reduced/increased depending on the flow rate of the fume hood). Quenching of intermediate morphologies was achieved by dialyzing a THF/water polymersome solution against water in a 3.5 kDa MWCO dialysis tubing after THF evaporation durations of either 3.5 h (for spherical polymersomes) or 5 h (for partially-faceted polymersomes). The water used for dialysis was replaced every 30 min for at least 4 h before being further dialyzed overnight.

Transmission Electron Microscopy (TEM)

TEM analyses were performed on an FEI Tecnai G2 20 TEM operating at an accelerating voltage of 200 kV. Images were acquired using a BM Eagle 2K CCD Camera. For aqueous polymersome samples, the grids were prepared by dropping 10 μL of sample (0.2 mg/mL) onto a formvar/carbon-coated copper grid. The droplet was left on the grid for at least 30 s before being blotted off with a filter paper, and then dried using a gentle stream of nitrogen. For polymersome samples containing THF, the grids were prepared by dropping 10 μL of sample (undiluted) onto a carbon only-coated grid. The droplet was immediately blotted off with a filter paper and dried using a gentle stream of nitrogen. No staining was used. Fast Fourier transform (FFT) analysis was performed using ImageJ.[2]

Cryogenic-Transmission Electron Microscopy (Cryo-TEM)

Cryo-TEM analyses were performed on an FEI Tecnai G2 20 TEM operating at an accelerating voltage of 200 kV. Images were acquired using a BM Eagle 2K CCD Camera and in-built low-dose software. Samples were vitrified using a Leica EM GP vitrification robot following a general procedure as follows: 3 μL of sample (1 mg/mL) was pipetted onto a 300 mesh copper grid with lacey formvar/carbon film support, and allowed to equilibrate for 30 s at room temperature and a relative humidity 90%. The sample droplet was then blotted from behind (1.8 s blot time; 1 s drain time) and plunged into liquid ethane held at -174 °C. Excess ethane was blotted away with a piece of pre-cooled filter paper. The vitrified grid was stored in a cryo-grid box immersed in liquid nitrogen before finally being cryo-transferred into the microscope using a Gatan 626 cryo-transfer holder.

Cryogenic-Electron Tomography (Cryo-ET)

As sample carriers, we used lacey carbon films on 300 mesh copper grids. Before usage, the grids were washed for 5 min in ethyl acetate and glow discharged for 30 s at 20 mA. For cryo fixation, 2 μL of

sample (1 mg/mL) was diluted with 15 nm protein-A gold fiducial markers at a volume ratio of 10:1, and frozen in liquid ethane at -180 °C using an FEI Vitrobot MK IV plunge freezer. Freezing conditions were 100% relative humidity at 22 °C, 3 µL sample application, 4.5 s blotting time at a blotting force of 10. Samples were stored in liquid nitrogen until further processing.

For the acquisition of tilt series, we used an FEI Tecnai F30 FEG-TEM equipped with a Gatan K2 Summit direct electron detector. The microscope was operated at 300 kV and images were recorded at a pixel size of 2.578 Å. As imaging acquisition software, we used SerialEM.[3,4] Samples were imaged at a temperature -179 °C under low-dose conditions over a tilt angle of +/-58° at 4° increments and a defocus of -2 µm. The total electron dose was 100 e/Å^2. For each tilt angle, 24 subframes were recorded in super-resolution mode, Fourier cropped in Serial EM to bin 1, and motion corrected with the program's frame alignment feature. For the tomographic reconstruction we used IMOD following the standard procedure for cryoEM including Cs correction and Nonlinear Anisotropic Diffusion filtering.[5,6]

Atomic Force Microscopy (AFM)

AFM measurements were performed on a Bruker Multimode 8 Atomic Force Microscope in Scanasyst (PeakForce Tapping®) mode. Bruker ScanAsyst-Fluid+ probes (spring constant, k = 0.7 N/m) were used. A typical sample preparation is as follows: a 5 µL drop of sample (undiluted) was placed onto a freshly cleaved mica substrate and smeared evenly using a glass slide. The resulting thin liquid film was then dried over a gentle stream of nitrogen before being imaged by AFM.

Dynamic Light Scattering (DLS)

DLS measurements were conducted on a Zetasizer Nano ZSP equipped with optical filters for improved measurement of fluorescent samples. All samples were prepared at a concentration of 0.1 mg/mL and measured at a backscatter angle of 173° in a quartz cuvette at 25 °C.

Fluorescence Spectroscopy

Fluorescence spectroscopy measurements were performed on a Varian Cary Eclipse Fluorescence Spectrophotometer. Samples were measured in a 0.7 mL dual pathlength (1.0/0.2 cm) quartz cuvette. The excitation source was applied perpendicular to the 1.0 cm path of the cuvette. The excitation and emission slit sizes were set to 10 and 5 nm, respectively. All samples were measured at a final concentration of 0.1 mg/mL in the desired THF/water mixture.

UV-Vis Spectroscopy

UV-Vis spectroscopy measurements were performed on a Varian Cary 60 UV-Vis Spectrophotometer. Samples were measured in a 0.7 mL dual path length (1.0/0.2 cm) quartz cuvette. All spectra were obtained across the 0.2 cm path length of the cuvette. Samples were measured at a concentration of 0.1 mg/mL in THF.

Supplementary Figures and Tables

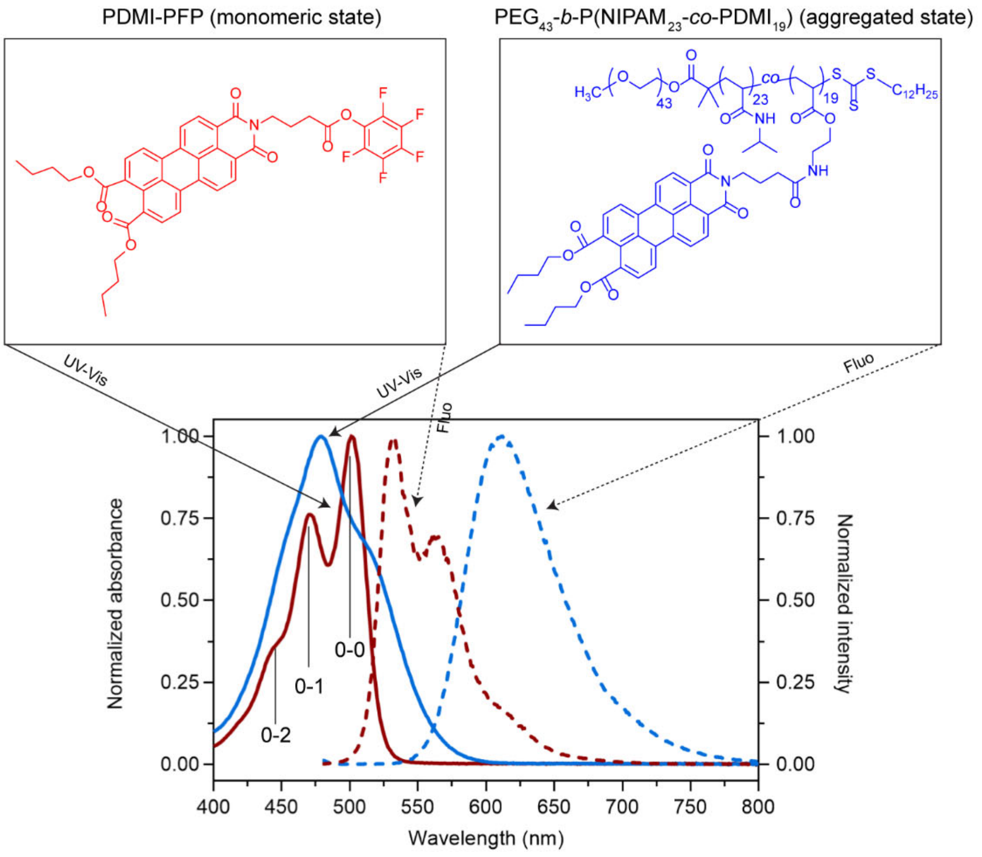

Figure S1. Normalized absorption (solid lines) and fluorescence spectra (dashed lines) of the pentafluorophenyl ester functionalized perylene diester monoimide PDMI-PFP (red) and PEG_{43}-*b*-P($NIPAM_{23}$-*co*-$PDMI_{19}$) (blue) in THF (0.1 mg/mL). Here, the data indicates that PDMI-PFP exists in a monomeric state while PEG_{43}-*b*-P($NIPAM_{23}$-*co*-$PDMI_{19}$) exists in an aggregated state in THF.

Additional discussion regarding Figure S1: Shown above are normalized absorption and emission spectra of 0.1 mg/mL THF solutions containing either (in red) pentafluorophenyl ester functionalized perylene diester monoimide PDMI-PFP and (in blue) the perylene-polymer PEG_{43}-*b*-P($NIPAM_{23}$-*co*-$PDMI_{19}$). In the absorption spectrum of PDMI-PFP (red solid line), three distinguishable vibronic peaks (corresponding to 0-0, 0-1 and 0-2 transitions) can be identified. These can be ascribed to vibrational modes that are coupled to the electronic S_0-S_1 transitions of the perylene chromophore. Since these vibronic peaks follow a normal Franck-Condon progression of $A_{0\text{-}0} > A_{0\text{-}1} > A_{0\text{-}2}$, PDMI-PFP can be interpreted to exist in a non-aggregated, monomeric state in THF.[7,8]

The perylene-polymer PEG_{43}-*b*-P($NIPAM_{23}$-*co*-$PDMI_{19}$) in THF, on the other hand, displays a broadened absorption profile with inverted intensities in the 0-0 and 0-1 vibronic peaks. This inversion in intensities, which can be further analyzed to give a $A_{0\text{-}0}/A_{0\text{-}1}$ value of 0.68 (typical of aggregated perylenes), is strong indication that the perylene moieties along a single polymer chain are aromatically

stacked in face-to-face fashion (H-aggregated).[8,9] This is further supported by the observation of a large red Stokes shift (132 nm) in the fluorescence spectra of PEG$_{43}$-*b*-P(NIPAM$_{23}$-*co*-PDMI$_{19}$) that strongly contrasts the mirror image relationship seen in the absorption and emission spectra of PDMI-PFP.[9–11] From our earlier work,[1] we know that extreme dilution, even down to a concentration of 0.017 mg/mL, does not help to disrupt the aggregated state of the perylene moieties due to strong intrachain aromatic stacking interactions.

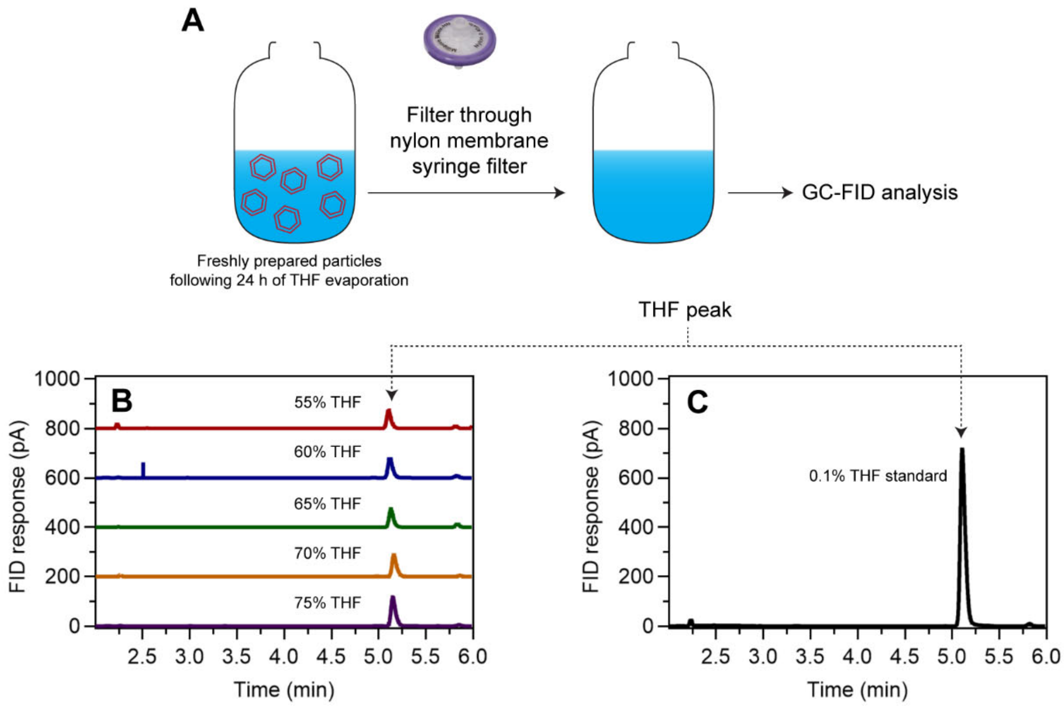

Figure S2. (A) Schematic illustration of the GC-FID sample preparation. (B) GC-FID traces of eluents collected from samples prepared at different %THFs following 24 h of THF evaporation. (C) GC-FID trace of a 0.1% THF standard solution.

Additional discussion regarding Figure S2: Shown above are gas chromatography-flame ionization detector (GC-FID) results which indicate that only very small amounts of THF remain in each sample after an evaporation time of 24 h. Briefly, in this experiment, we first removed the polymer particles in each sample using nylon membrane syringe filters (Figure S2A), collected individual eluents, and quantified any remaining THF in each eluent via GC-FID. Quantitative analysis of the data (see Table S1) revealed that less than 0.015% (v/v) of the original amount of THF used remains in all samples following an evaporation time of 24 h. From earlier work,[1] we know that this rather small amount of THF can also be effectively removed via dialysis against water.

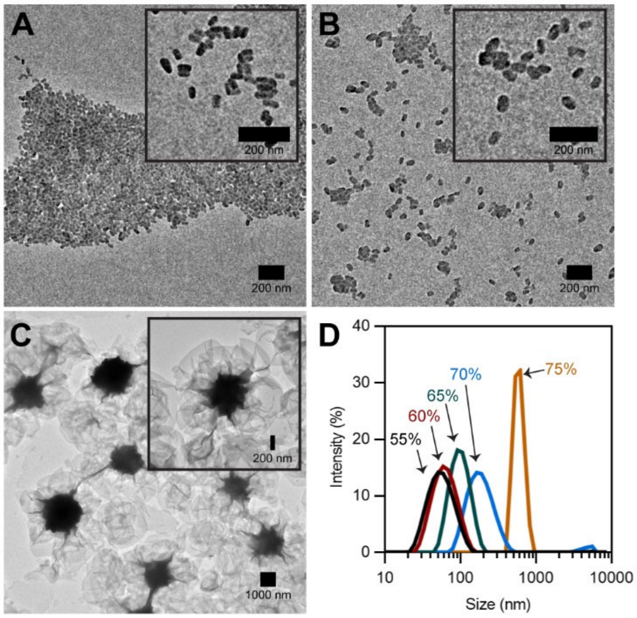

Figure S3. TEM images of PEG$_{43}$-*b*-P(NIPAM$_{23}$-*co*-PDMI$_{19}$) self-assembled structures prepared using (A) 55%, (B) 60%, (C) 75% THF/water. (D) Particle size distribution of all five morphologies in aqueous solution obtained using dynamic light scattering (DLS).

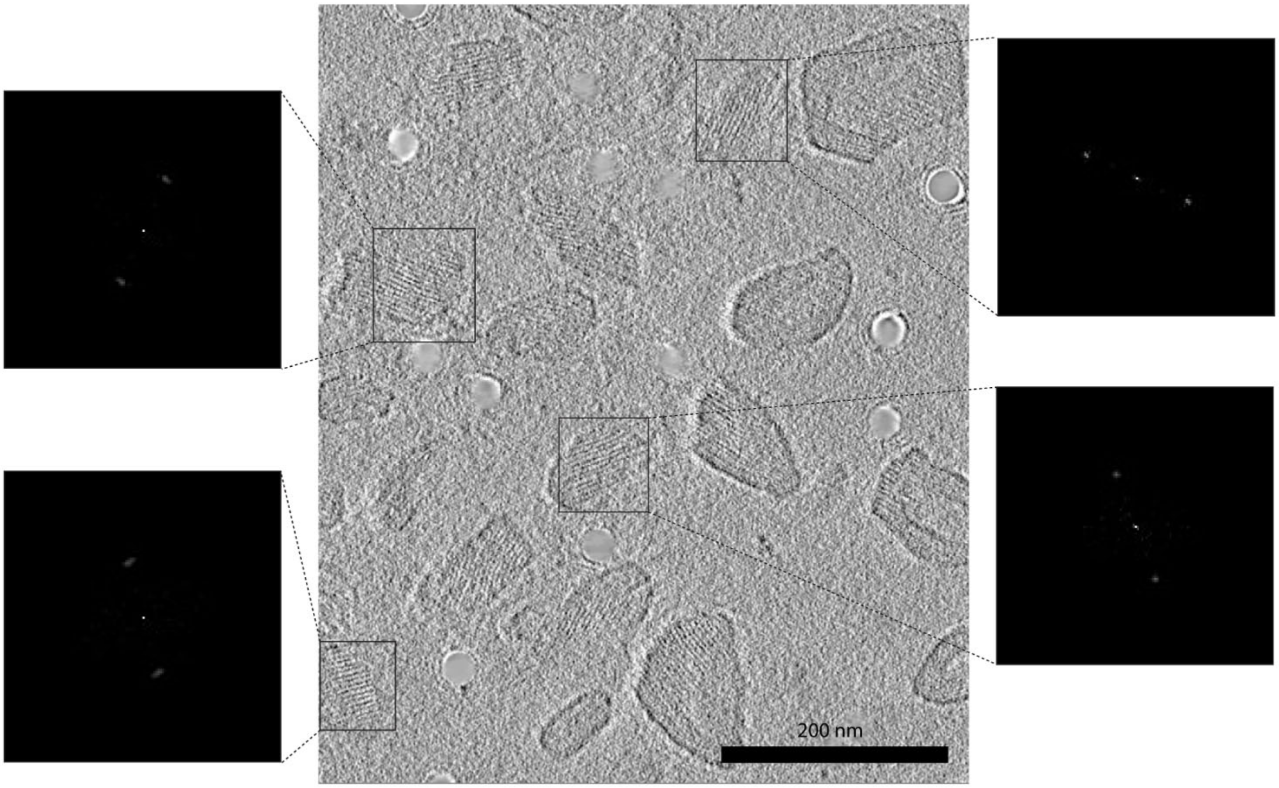

Figure S4. Four fast Fourier transform (FFT) patterns obtained from different regions of interest of the cryo-ET image shown in Figure 3F. All FFT spots correspond to a *d*-spacing of 2.2 nm.

Table S1. Integrated THF peak areas from the GC-FID traces shown in Figure S2. The relative peak areas were used to estimate the amount of residual THF in each sample after an evaporation time of 24 h.

Sample	Peak area (counts)	Peak area (rel. to standard)	%THF in water	V_{THF}[a] in 1 mL water[b] (μL)	V_{THF} used to prepare particles (μL)	%THF left (rel. to V_{THF} used)
55% THF	183	11.3	0.009	0.089	611	**0.0145**
60% THF	213	9.7	0.010	0.104	750	**0.0138**
65% THF	183	11.3	0.009	0.089	929	**0.0096**
70% THF	231	8.9	0.011	0.112	1167	**0.0096**
75% THF	304	6.8	0.015	0.148	1500	**0.0098**
Standard[c]	2057	1.0	0.100	1.000	-	-

[a]V_{THF} = volume of THF; [b]All samples were diluted to 1 mL with water prior to filtration and analysis; [c]Standard = 0.1% THF in water

Table S2. Solvent mixtures used in self-assembly and DLS results.

Sample	V_{THF}[a] (μL)	V_{water}[b] (μL)	D_h[c] (nm)	PDI[d]
55% THF	611	500	51 ± 0.3	0.197 ± 0.008
60% THF	750	500	56 ± 0.7	0.164 ± 0.022
65% THF	929	500	93 ± 1.5	0.075 ± 0.014
70% THF	1167	500	180 ± 3.2	0.202 ± 0.005
75% THF	1500	500	782 ± 73	0.380 ± 0.135

[a]V_{THF} = volume of THF; [b]V_{water} = volume of water; [c]D_h = *Z*-average hydrodynamic diameter; [d]PDI = polydispersity index.

References

1 C. K. Wong, A. F. Mason, M. H. Stenzel and P. Thordarson, *Nat. Commun.*, 2017, **8**, 1240.

2 C. A. Schneider, W. S. Rasband and K. W. Eliceiri, *Nat. Methods*, 2012, **9**, 671–675.

3 D. Mastronarde, *Microsc. Microanal.*, 2003, **9**, 1182–1183.

4 D. N. Mastronarde, *J. Struct. Biol.*, 2005, **152**, 36–51.

5 J. R. Kremer, D. N. Mastronarde and J. R. McIntosh, *J. Struct. Biol.*, 1996, **116**, 71–6.

6 D. N. Mastronarde and S. R. Held, *J. Struct. Biol.*, 2017, **197**, 102–113.

7 W. Wang, J. J. Han, L.-Q. Wang, L.-S. Li, W. J. Shaw and A. D. Q. Li, *Nano Lett.*, 2003, **3**, 455–458.

8 C. Shao, M. Grüne, M. Stolte and F. Würthner, *Chem. Eur. J.*, 2012, **18**, 13665–13677.

9 G. Echue, G. C. Lloyd-Jones and C. F. J. Faul, *Chem. Eur. J.*, 2015, **21**, 5118–5128.

10 F. Fennel, S. Wolter, Z. Xie, P. A. Plötz, O. Kühn, F. Würthner and S. Lochbrunner, *J. Am. Chem. Soc.*, 2013, **135**, 18722–18725.

11 S. Rehm, V. Stepanenko, X. Zhang, T. H. Rehm and F. Würthner, *Chem. Eur. J.*, 2010, **16**, 3372–3382.
